# Knockdown of LRP5 Promotes Proliferation and Invasion of Tongue Squamous Cell Carcinoma through Compensatory Activation of Akt Signaling

**DOI:** 10.7150/jca.93585

**Published:** 2024-04-15

**Authors:** Chengze Wang, Yongzheng Li, Xiaoyan Miao, Ying Wang, Guoli Yang

**Affiliations:** Stomatology Hospital, School of Stomatology, Zhejiang University School of Medicine, Clinical Research Center for Oral Diseases of Zhejiang Province, Key Laboratory of Oral Biomedical Research of Zhejiang Province, Cancer Center of Zhejiang University, Hangzhou 310006, China.

**Keywords:** LRP5, Akt signaling pathway, Tongue squamous cell carcinoma, Invasion, Wnt pathway

## Abstract

The role of LRP5, a critical receptor in the Wnt signaling pathway, remains unexplored in tongue squamous cell carcinoma (TSCC). This study investigates the impact of LRP5 knockdown on the biological behaviors of TSCC cell lines both *in vitro* and *in vivo*. Our findings indicate that LRP5 knockdown significantly enhances cell proliferation, migration, and invasion in CAL27 and SCC25 cell lines. RNA-seq analysis reveals compensatory activation of the Akt pathway, with 119 genes significantly upregulated post-LRP5 knockdown. Elevated MMP1 expression suggests its potential involvement in TSCC progression. Western blot analysis demonstrates increased Akt phosphorylation, upregulated proliferation-related PCNA, and downregulated apoptosis-related caspase-3 after LRP5 knockdown. Down-regulation of E-cadherin and β-Catenin, proteins associated with cell adhesion and invasion, further elucidates the molecular mechanism underlying increased cell migration and invasion. Our study concludes that compensatory Akt pathway activation is essential for the LRP5 knockdown-induced migration and proliferation of CAL27 and SCC25 cells. These results highlight LRP5 as a potential therapeutic target for TSCC. Simultaneous inhibition of Wnt and Akt signaling emerges as a promising approach for TSCC treatment.

## Introduction

Tongue squamous cell carcinoma is one of the most common cancers in the neck and head 1, which often results in short survival and poor prognosis, even after surgery and chemotherapy. Cell surface molecules can be used as good targets for anti-tumor drugs, such as epidermal growth factor receptor (EGFR), PD-L1, and insulin-like growth factor 1, all of which have been successfully used in the clinical treatment of tumors. It is much harder for resistant cells to develop resistance against surface receptor molecules than drug molecules. LRP5 (low-density lipoprotein receptor-related protein 5) is a transmembrane protein, as a co-receptor of the WNT signaling pathway. With the co-receptor's LRP5/6, the family of Frizzled (FZD) receptors interacts with WNT ligands to activate the canonical WNT/β-catenin signaling cascade. Deregulation of this pathway is involved in the progression, drug resistance, and immunity escape of several cancers 2-4. Therefore, LRP5 may be an ideal cell surface molecular target to regulate the Wnt signaling pathway in the treatment of head and neck squamous cell carcinoma.

The WNT signaling pathway is crucial in both head and neck cancer, including TSCC, where its dysregulation contributes to tumor initiation, progression, and metastasis. Specifically in TSCC, abnormal activation of WNT signaling fosters tumor cell proliferation, invasion, and epithelial-mesenchymal transition (EMT), resulting in aggressive tumor behavior and unfavorable prognosis 5,6. The interaction between Wnt and Frizzled (Frz) activates the noncanonical pathway, while the activation of the canonical pathway requires the involvement of Wnt co-receptors LRP5/6. The canonical Wnt1 gene was initially discovered as a preferred integration site for the mouse mammary tumor virus in virally induced breast tumors. Subsequently, LRP5/6 were found to play a crucial role in Wnt1-dependent tumor development in transgenic mouse models 7-9. In preliminary experiments, we found that elevated expression of LRP5 in TSCC may be associated with patient prognosis. Thus, components of the canonical pathway, including LRP5/6, are commonly implicated in cancer progression as oncogenes. LRP5/6's ectodomain comprises four β-propeller/epidermal growth factor (EGF) repeats (E1-4) and three LDL repeats (LDLR), with E1-4 serving as the binding domain for canonical Wnt ligands and the canonical pathway inhibitor Dkk1^10^-^13^. However, the proteins binding to LDLR remain unexplored. Activation of the canonical pathway requires the close proximity of LRP5/6 and Frz, facilitated by canonical Wnt ligand binding to E1-4 of LRP5/6 and the amino-terminal cysteine-rich domain (CRD) of Frz^14^,^15^. Conversely, Dkk1 induces LRP5/6 internalization via its receptor Kremen, preventing Wnt reception and inhibiting the canonical pathway. Key components like LRP5 act as co-receptors, transmitting WNT signals and facilitating oncogenic processes across various cancers 16-18. Understanding the complex role of WNT signaling in head and neck cancer highlights its significance as a potential therapeutic target against this formidable disease.

For the role of LRP5 in tumor cells, there are two types of research results in the past. The first type is to knockdown the expression of LRP5 through siRNA or shRNA lentivirus, and it is found that knockdown the expression of LRP5 can inhibit the growth of tumor cells 19. Or by overexpressing the dominant-negative, soluble LRP5 (sLRP5) can reverse epithelial-mesenchymal transition of tumor cells by blocking Wnt signaling 20-22. Activation of LRP5 gene promotes CSCs-like phenotypes, including tumorigenicity and drug resistance in colorectal cancer cells 23. Interestingly, some studies have shown that LRP5 knockdown does not slow the proliferation and invasion of certain cells, and the mechanism is not very clear. Ren 24 has shown that in breast cancer, knockdown of LRP5 or LRP6 suppresses the Wnt signaling, but it increases lung metastasis in breast cancer cells in nude mice. Maria and her colleagues 25 find that LRP5 overexpression inhibits cell proliferation and induces apoptosis in HL60 cells. Some scholars 26 chose to examine the effects of LRP5 mRNA expression on patient survival in six different malignancies, including colorectal adenocarcinoma, and failed to demonstrate a significant effect. In this study, we wanted to study the effects of LRP5 knockdown on the biological behavior of tongue cancer cell lines and their possible mechanisms.

According to previous studies, the activation of canonical Wnt/β-catenin signaling promotes invasion, proliferation, and anti-apoptosis in TSCC cells 27-30. Targeted inhibition of the Wnt signaling pathway is considered promising in the treatment of TSCC and even head and neck cancer. Certain inhibitors of the β-catenin complex or neutralizing antibodies to FZD/LRP receptors have been gradually applied in some clinical trials. ICG-001 and the second-generation compound PRI-724 that selectively inhibit the CBP/β-catenin complex are used in clinical trials in patients with certain solid tumors 31. Vantictumab 32, an anti‑Frizzled‑1/2/5/7/8 antibody, is also being used in clinical trials for certain solid tumors.

Our study found that after LRP5 knockdown in different tongue cancer cell lines, the growth of tumor cells was accelerated. Transcriptome sequencing found that after CAL27 and SCC25 were transfected with siLRP5 lentivirus, the Akt signaling pathway was abnormally activated. Compensatory activation of the Akt signaling pathway resulted in faster cell growth. We speculate that on the basis of inhibiting the Wnt signaling pathway, combined with inhibiting the activation of the Akt signaling pathway, the dual inhibition may limit the growth and progression of tongue cancer cell lines *in vitro* and *in vivo*.

## Materials and methods

### Antibodies and reagents

The LRP5 (#5731), p-Akt (#4060), pan-Akt (#4685), and E-cadherin (#14472) antibodies are all manufactured by Cell Signaling Technologies (Beverly, MA, USA). The p-mTOR (AP0115), mTOR (A2445), β-Actin (AC026), and PCNA (A12427) were purchased from Abclonal (China). The β-Catenin (51067-2-AP) and HRP-conjugated GAPDH (HRP-60004) are from Proteintech. The cleaved caspase-3 (ab32351) antibody is bought from Abcam (Cambridge, UK). The Akt inhibitors included GSK690693, AZD5363 and LY294002 and crystal violet are all manufactured by Beyotime (China). The alamarBlue is from thermofisher (Cleveland, OH, USA).

### Cell Culture

The CAL27 (CVCL_1107) and SCC25 (CVCL_1682) cells were cultured in high glucose Dulbecco's modified Eagle's medium (DMEM; Gibco, Invitrogen) with 10% fetal bovine serum (FBS; GEMINI) and 1% penicillin-streptomycin. Cells were purchased from Shanghai Zishi Biological Company and identified by STR. All experiments were performed using mycoplasma-free cells. Cells were incubated in a humidified atmosphere at 37°C with 95% air and 5% CO2. The siLRP5 and siNC lentiviruses were synthesized by Gcbio (Shanghai, China) and stably transfected after puromycin (1ug/ml) was added for 7 days. The alamerBlue assay procedure was conducted. First, cells were seeded into a 96-well plate at 1000 cells per well. After allowing the cells to attach and proliferate for a specified time, 10 μl alamerBlue was added to each well. Next, the plate was incubated for an hour. The results were then quantified by measuring the absorbance of the sample at 570 nm.

### Wound healing assay

Cells were cultured to about 80% confluence before FBS starvation for 24 h. A 200 μl conventional pipette tip was used to create a scratch wound across the cell layer, followed by rinsing with PBS twice. Thereafter, culture was conducted in 1% FBS-containing DMEM. The wound width was evaluated via Image-Pro Plus 6.0 software after 20h.

### Protein extraction and western blot analysis

Cells were washed twice with cold PBS and lysed with RIPA buffer containing PMSF at 4°C for 30 min. Then, the cells were collected and centrifuged at 4°C and 12,000 rpm for 15 min. For western blot assay, 20 μg of protein was isolated on 10% SDS-polyacrylamide gels, and transferred onto polyvinylidene difluoride (PVDF) membranes (Millipore, Bedford, MA, USA). The membranes were then blocked with TBST containing 5% fat-free milk, and incubated overnight at 4°C with primary antibodies against p-Akt (Ser473; 1:1000), pan-Akt (1:1000), LRP5 (1:1000), p-mTOR (S2448; 1:1000), mTOR (1:1000) , caspase-3 (1:500) , E-caderin (1:1000), β-Catenin (1:1000), PCNA (1:1000), Beta-Actin (1:1000), and GAPDH (1:1000). The membranes were then washed with TBST, and incubated with HRP-conjugated secondary antibodies (1:4,000) for 1 h at room temperature. After being washed three times with TBST, the membrane was subjected to ECL detection using Bio-Rad chemiluminescence system (Bio-Rad, USA). Finally, the band densities were analyzed using ImageJ software (National Institutes of Health, Bethesda, MD, USA).

### Colony formation assay

For colony formation assay, Cells were plated at 500 cells per well in six-well plates in DMEM medium plus 10% FBS. Cells were replaced with fresh medium every two to three days. After 8 days, colonies were fixed with paraformaldehyde, stained with crystal violet, imaged and counted under an inverted microscope. Each colony contains more than 100 cells. The data represents means ± standard error of three independent wells.

### Animal model

All animal protocols were approved by the Animal Care and Use Committee at the Zhejiang University School of Medicine and followed the NIH Guidelines for Care and Use of Animals in Research (NIH Publication No. 85-23, revised 1996). Female BALB/c nude mice at 4 weeks old were purchased from Zhejiang Academy of Medical Sciences and fed with standard rodent chow under specific pathogen-free (SPF) conditions. Mice were given a subcutaneous injection of a mixture containing 2 × 10^6^ CAL27 or 1 × 10^6^ SCC25 cells, with each cell resuspended in 150 μl of PBS. Mice were anaesthetized 30 days later and sacrificed to harvest all tumors. The tumor weight was measured. For the mouse model of lung metastasis, female BALB/c nude mice at 4 weeks old were used. About 1 × 10^6^ cells in 100 ul PBS were injected into mice through the tail vein. One months later, the left and right lungs of each animal were fixed in 4 % paraformaldehyde and sections were stained with haematoxylin and eosin (H&E) for histology examination.

### Immunofluorescence assay

For immunofluorescence assay, cells were seeded on glass coverslips in a 6-well plate and incubated for 48 hours. Then, cells were fixed with paraformaldehyde for 15 minutes at room temperature and washed with PBS. After a blocking step with normal goat serum for 60 min at room temperature, cells were incubated with associated antibodies (E-cad, 1:100; LRP5, 1:200; p-Akt, 1:200) overnight at 4°C. Subsequently, slides were washed with PBS three times and incubated with DyLight 488 or DyLight 594 second antibody for 1 hour at room temperature. Following a double PBS wash, slides were stained with DAPI (4',6-Diamidino-2-Phenylindole) and examined with a fluorescence microscope.

For immunohistochemistry assay, the tumor tissues from the mice subcutaneous xenograft model were fixed with paraformaldehyde for 24 hours, embedded in paraffin and then sectioned (4 μm) using a RM2135 rotary microtome (Leica Geosystems). The sections were subjected to a series of preparatory treatments: roasted at 60◦C for 2 hours, dewaxed with dimethyl benzene, dehydrated with alcohol, and antigen retrieval was performed with a solution. The sections were then incubated with anti-p-Akt primary antibody (diluted 1:200) overnight at 4◦C following 1 × TBST washing. After 1 × TBST washing, the sections were further incubated with a biotinylated secondary antibody for 1 h before being washed and counterstained with hematoxylin (Sigma-Aldrich). Imaging was conducted using an inverted fluorescence microscope.

### Bioinformatics analysis

After mRNA was extracted from CAL27 and SCC25 cells stably transfected with siLRP5 and siNC lentiviruses, followed by reverse transcription to generate cDNA, and finally the sequencing of the cDNA was obtained. After quality control and filtering of the raw sequencing data by Fastp software, the data was compared to the reference genome using Hisat2. Then the gene reads data is converted into a standardized TPM. The probe sets without corresponding gene symbols or the genes with multiple probe sets were removed or averaged respectively. The DESeq R package was used to obtain DEGs with MARS algorithm 33. Log2FC> 1 or Log2FC< - 1, p-value < 0.05 were considered to indicate statistical significance. At last, the common up-regulated genes and down-regulated genes between different groups were selected. We performed gene ontology (GO) and Kyoto Encyclopedia of Genes and Genomes (KEGG) analysis of common DEGs (119 up-regulated genes) between the siNC and siLRP5 groups. GO and KEGG enrichment analyses were performed via an R package called clusterProfiler^34^. The analyses were done by enrichGO and enrichKEGG functions, respectively 35. The Pathview library of the bioconductor was used to generate the Akt signaling pathway. The fold values of significantly changed genes were mapped by colors on native KEGG, Akt signaling pathway, where green represents down-regulated expression and red represents up-regulated expression levels in relation to the control group. A p value < 0.05 was considered significant. The data correlating MMP1 expression levels with the survival prognosis of head and neck squamous cell carcinoma patients was obtained from GEPIA 36. The expression data of AKT1 was obtained from proteinatlas.org and analyzed using R language, utilizing the survminer and survival packages for survival analysis curve plotting. The AKT1 expression data is included in the supplementary files.

### Statistical analysis

All statistical analyses were performed using SPSS version 19.0 (SPSS, Chicago, IL). Student's t-test or Analysis of Variance was used to compare group distributions. All results were expressed as mean ± standard deviation (S. D.). A value of P less than 0.05 was considered statistically significant.

## Results

### Knockdown of LRP5 promotes tongue squamous cell carcinoma CAL27 and SCC25 proliferation and migration *in vitro*

**Figure 1:**
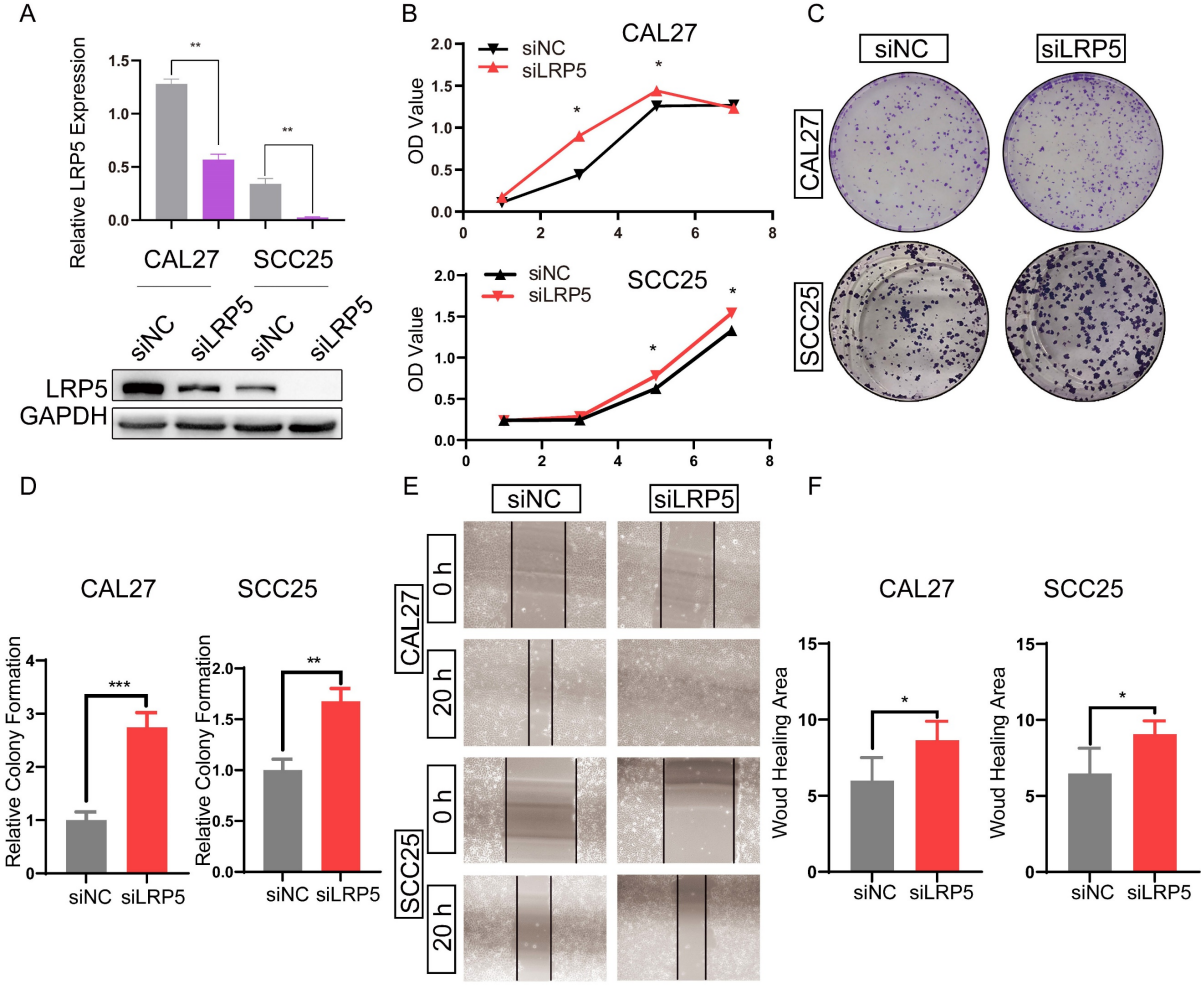
** LRP5 knockown promoted the proliferation and migration of CAL27 and SCC25 cells.** (A) Western blot assay showed the expression of LRP5 was down-regulated after cells were transfected with lentivirus and selected with 1 µg/ml puromycin for 7 days. (B and C) Cell proliferation was measured by alamarBlue and colony formation assays. (D) The relative value of colony formation compared to the siNC group was indicated. (E and F) Cell migration was measured by wound healing assay. Cell-based experiments were repeated three times with triplicate wells each. (*P <0.05, **P <0.001, *** P <0.0001)

To study the effect of LRP5 knockdown on tongue squamous cell carcinoma, LRP5 siRNA lentivirus was used to transfect CAL27 and SCC25. We evaluated the effect of LRP5 knockdown on biological behaviors of tongue squamous cell carcinoma cell line CAL27 and SCC25. The efficiency of knockdown of LRP5 was evaluated by Western blot assay. Results of Western blot assay showed that LRP5 expression was significantly down-regulated (Fig. 1A). Following knockdown of LRP5, increased cell proliferation was observed in Alamarblue and colony formation assays. (Fig. 1B-D) Moreover, cell migration significantly elevated in the wound healing assay (Fig. 1E, F).

### LRP5 knockdown promotes CAL27 and SCC25 proliferation and invasion *in vivo*

**Figure 2:**
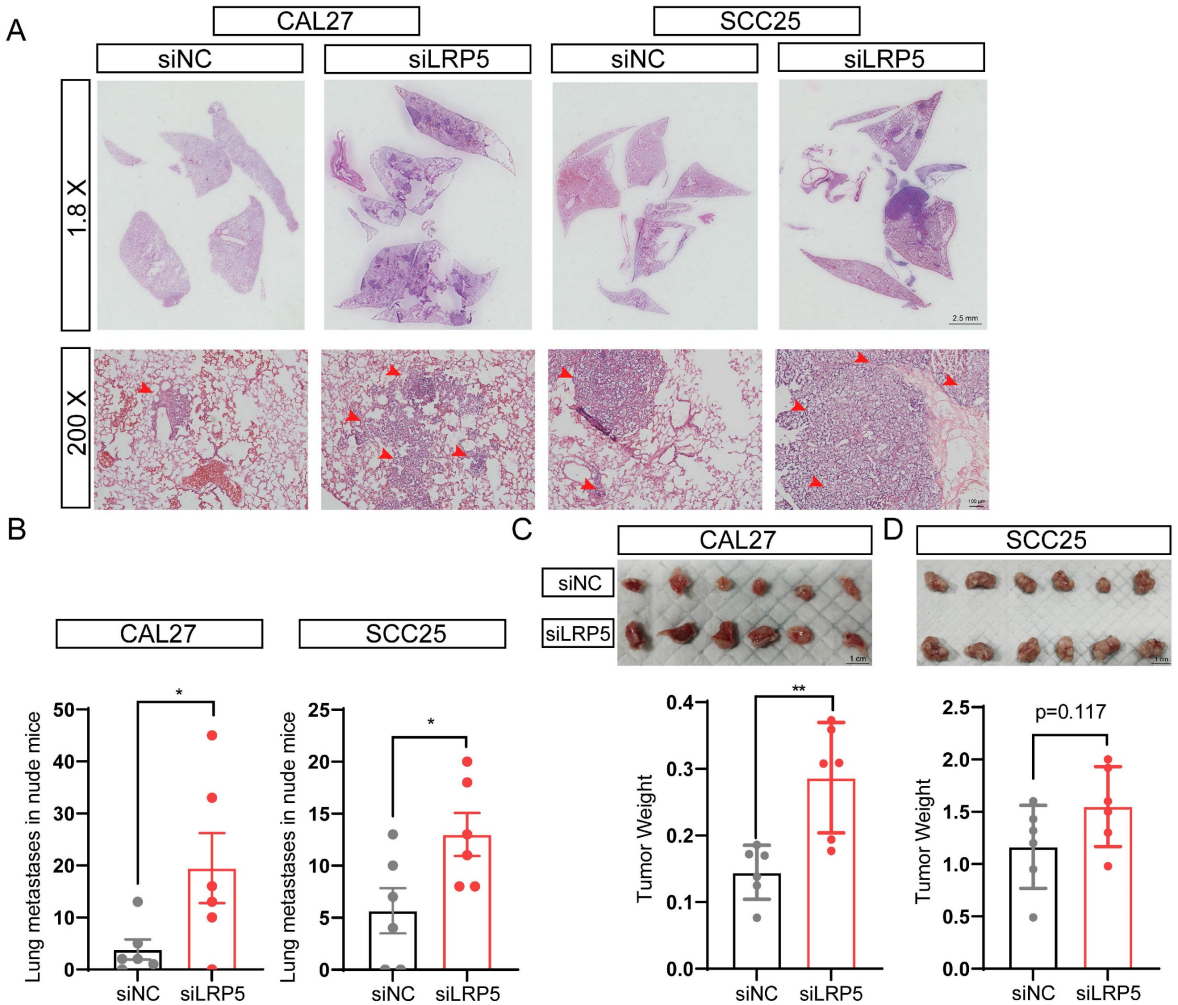
** LRP5 knockown promoted the proliferation and invasion of CAL27 and SCC25 cells *in vivo*.** (A and B) About 1 × 10^6^ cells in 100 ul PBS were injected into female BALB/c nude mice through the tail vein (the siLRP5 group, n = 6; the siNC group, n = 6). One months later, the haematoxylin and eosin staining of lung paraffin sections showed that siLRP5 group had more lung metastasis nodules than siNC group in both CAL27 and SCC25 cells. The red arrows indicate tumor cells metastasized to the lungs. (C and D) Tumor xenograft model in nude mice indicated that the siLRP5 group had larger subcutaneous tumor formation than the siNC group. (*P <0.05, **P <0.001)

The lung metastasis nude mice model and subcutaneous tumor model were established, the proliferation and invasion ability of tumor cells *in vivo* was evaluated after LRP5 knockdown. The results showed that knockdown of LRP5 increased the metastatic nodules of lungs (Fig. 2A, B). The hematoxylin and eosin staining of lung slides also suggested a marked increase in the area of lung metastasis in the siLRP5 group (Fig. 2A). Compared to those of control xenografts, LRP5 knockdown accelerated the tumor growth in nude mice by weight over a period of 30 days in the subcutaneous tumor model. (Fig. 2C, D).

### Akt pathway is activated by LRP5 knockdown

**Figure 3:**
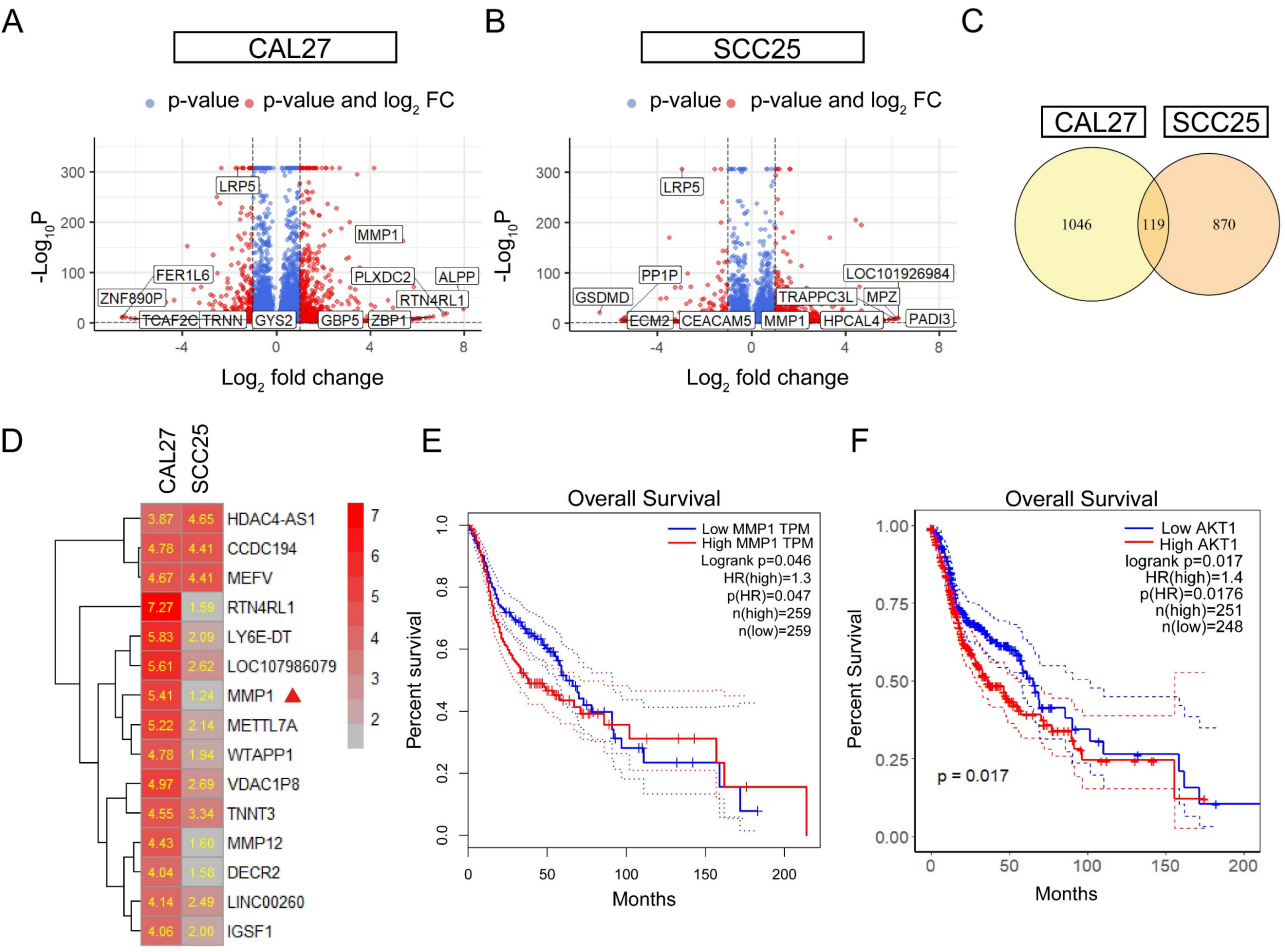
** MMP1 and Akt pathway were induced in the siLRP5 group than the control group.** CAL27 and SCC25 in LRP5 knockdown group and siNC group were analyzed by transcriptome sequencing separately. (A and B) The volcano map showed differentially expressed genes (DEGs) after knockdown of LRP5 in two kinds of cells. (C) The venn map showed that 1165 genes were upregulated in CAL27 and 989 genes were upregulated in SCC25 after knockdown of LRP5. There were 119 identical genes among them. (D) The heatmap of DEGs showed that MMP1 was elevated (Mark with a red triangle). (E) According to the GEPIA website, patients with high expression of MMP1 in head and neck squamous cell carcinoma have a lower likelihood of survival. (F) According to public data from the HPA website (supplementary files provide the raw data for analysis), R language analysis revealed that patients with high expression of AKT1 in head and neck squamous cell carcinoma have a worse prognosis relative to patients with low expression of AKT1.

**Figure 4:**
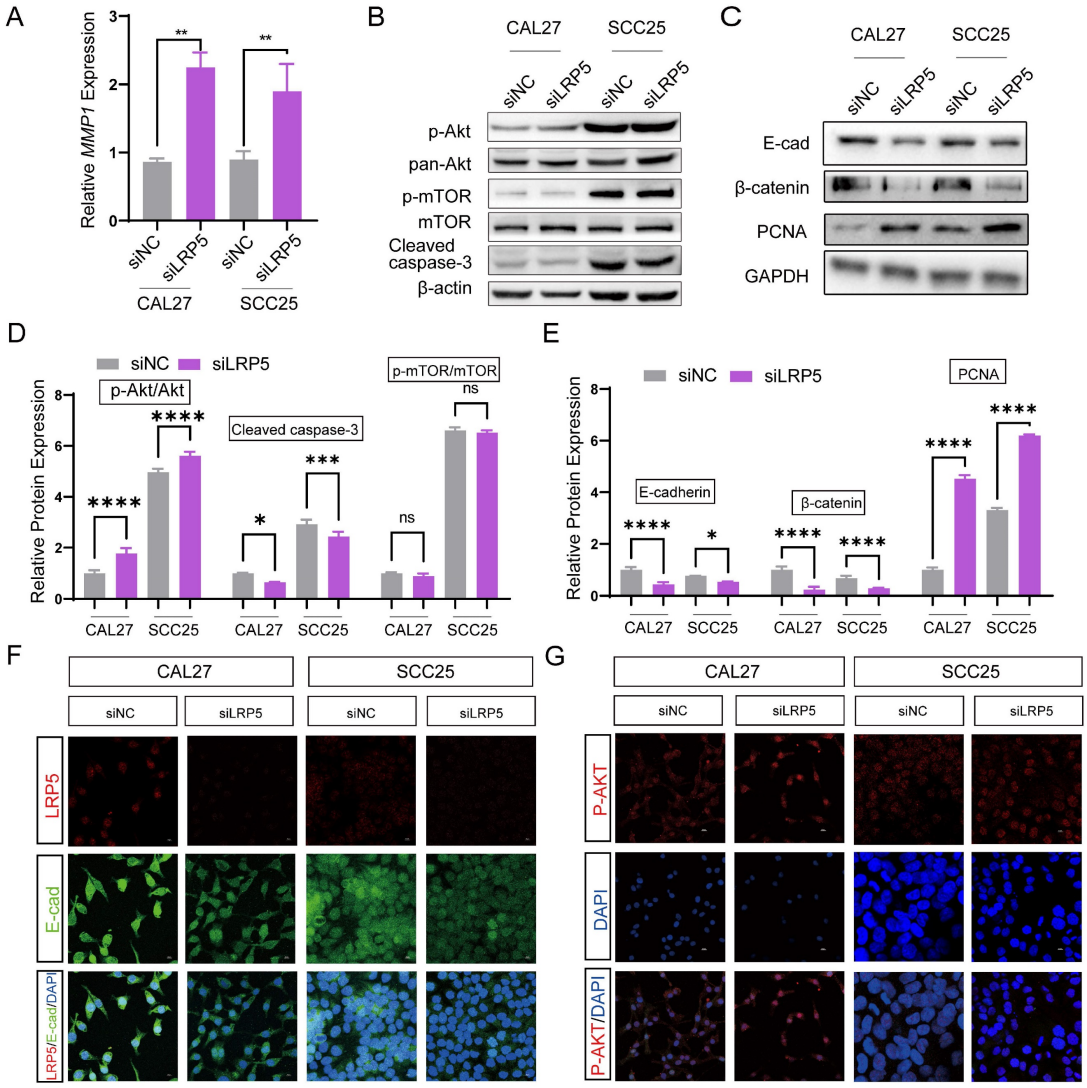
** LRP5 knockdown induced compensatory activation of the Akt signaling pathway.** (A) Quantitative PCR verified that *MMP1* increased after LRP5 knockdown in both CAL27 and SCC25 cell lines. (B and D) After knockdown of LRP5, p-Akt expression significantly increased. In addition, the expression of apoptosis-related protein cleaved caspase-3 decreased. (C and E) Western blot showed that the expression of the cell cycle-related protein PCNA increased, which verified the accelerated cell proliferation. In addition, the expression of adhesion-related proteins E-cad and β-catenin decreased. (F and G) By immunofluorescence staining, in the siLRP5 group, the cells had less E-cad fluorescence, but stronger fluorescence expression of p-Akt compared to the siNC group. ( scale bar = 10 μm)

**Figure 5:**
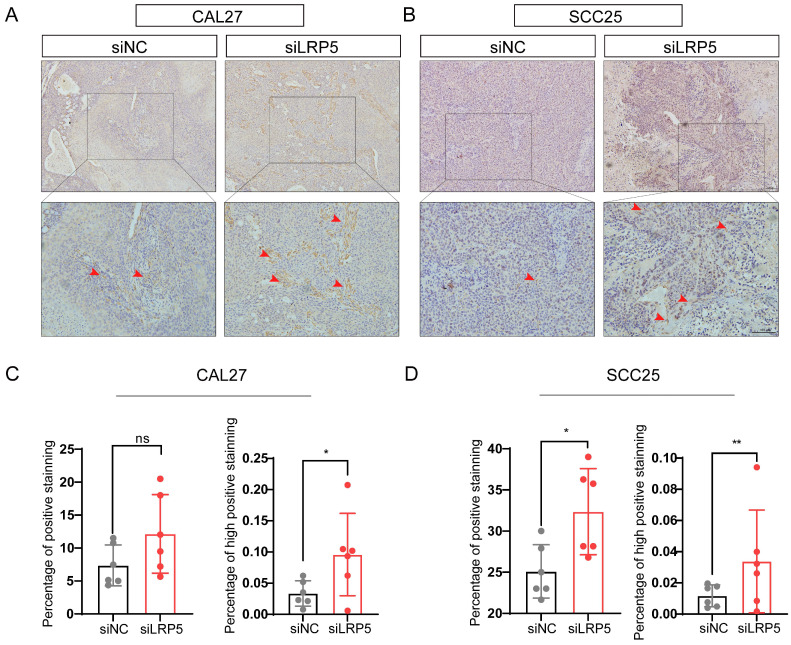
** P-Akt expression increased *in vivo* in the tumor xenograft model after LRP5 knockdown.** Mice were given a subcutaneous injection of 2 × 10^6^ CAL27 or 1 × 10^6^ SCC25 cells (the siLRP5 group, n = 6; the siNC group, n = 6), with each cell resuspended in 150 μl of PBS. Mice were anaesthetized 30 days later and sacrificed to harvest all tumors. (A and B) Immunohistochemical staining of paraffin sections of tumor xenograft tissue showed increased intensity and regions of ​​p-Akt expression. The red arrow indicates tumor cells with high expression of p-AKT. (C and D) The intensity and expression regions of p-Akt were analyzed using the plugin IHC Profiler for ImageJ. The results of the statistical analysis were displayed in histograms. (*P <0.05, **P <0.001)

To further investigate the mechanisms of LRP5 knockdown, RNA-seq was performed. The differentially expressed genes (DEGs) after LRP5 knockdown were obtained (logFC = 1, p < 0.05) (Fig. 3A, B). Then DEGs of the two cell lines CAL27 and SCC25 were crossed, and 119 up-regulated DEGs (Table S1) were obtained after LRP5 knockdown (Fig. 3C). Using functional enrichment analysis, it was obtained that the Akt signaling pathway was compensatory activated compared to the control group in both cell lines (Fig. S1). Using bioinformatic tools, we found 25 genes that were significantly upregulated in both cell lines. The heatmap displays 15 representative commonly upregulated genes (Fig. 3D). It has been reported in the literature that the expression of MMP1 is increased in tongue squamous cell carcinoma and is associated with the survival and prognosis of patients 37-39. They 37 found that knocking down MMP1 can reduce the proliferation and invasion of the tongue squamous cell carcinoma cell line FADU, and also lead to decreased phosphorylated Akt. Additionally, we have analyzed the correlation between MMP1 expression levels and the prognosis of head and neck squamous cell carcinoma patients through database analysis (with tongue squamous cell carcinoma comprising the majority, approximately 25.2%, from GDAC Firehose). We found that high expression of MMP1 and AKT1 is associated with lower survival chances in patients with head and neck squamous cell carcinoma compared to low expression (Fig. 3E, F). PCR verified that MMP1 was indeed elevated after LRP5 knockdown (Fig. 4A). The results of western blot assay showed that the phosphorylation of Akt was increased, the expression of proliferation-related PCNA was increased, and the apoptosis-related protein caspase3 was decreased (Fig. 4B-E). We also found that the protein E-cadherin and β-Catenin related to cell adhesion and invasion were down-regulated, which further proved the molecular mechanism of increased cell migration and invasion (Fig. C, E). Furthermore, in immunofluorescence staining assay, we observed that the fluorescence intensity of p-Akt increased after LRP5 knockdown, while that of E-cadherin decreased (Fig. 4F, G). In the *in vivo* subcutaneous tumor xenografts model, the intensity of p-Akt immunohistochemical staining was significantly greater in the siLRP5 group than in the control group (Fig. 5A-D).

### Compensatory Akt activation is required for LRP5 knockdown-induced cell proliferation and migration

**Figure 6:**
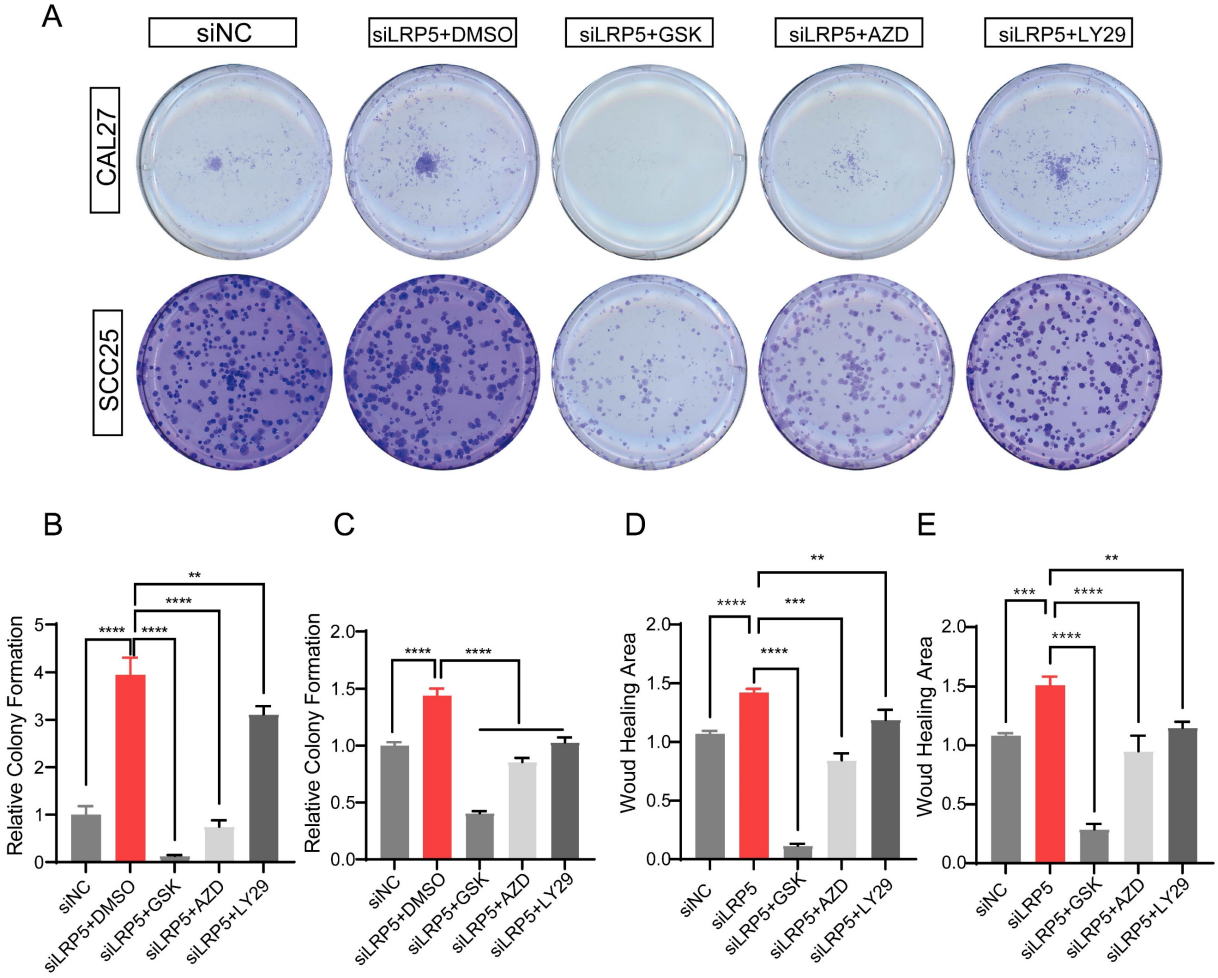

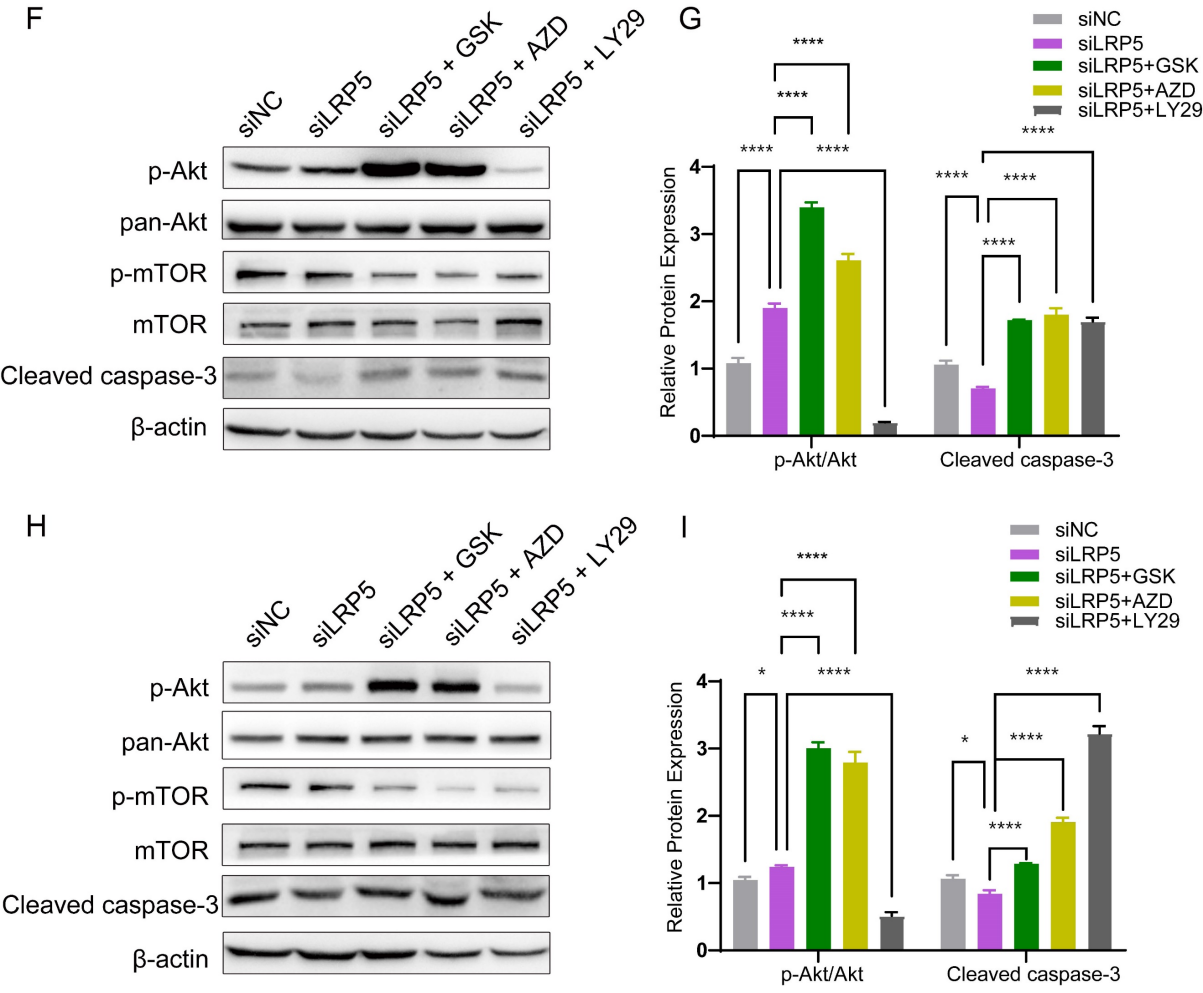
** Akt inhibitors inhibited cell proliferation and migration caused by knockdown of LRP5.** (A-C) About 500 cells were plated in six-well plates. Akt inhibitors (LY294002 = 1 μM; GSK690693 = 1 μM; and AZD5363 = 1 μM) or DMSO were added to the siLRP5 group and cultured for 3 days, and then replaced with normal complete medium without inhibitors. 5 days later, the cells were fixed and stained with crystal violet. Akt inhibitors can significantly inhibited the colony formation of tumor cells after LRP5 knockdown. Among them, GSK690693 showed the most obvious inhibitory effect, and LY294002 had the worst effect. (D and E) Cells in different groups were pretreated with DMSO or Akt inhibitors (LY294002 = 1 μM; GSK690693 = 1 μM; and AZD5363 = 1 μM) for 3 hours. Then, wound healing experiments were carried out. Akt inhibitors also inhibited the migration of tumor cells after LRP5 knockdown by wound healing assay. (F-I) Western blot showed that Akt inhibitors increased the expression of cleaved caspase-3 and inhibited the expression of p-mTOR. Interestingly, GSK690693 and AZD5363 increased p-Akt accumulation in cells. However, LY294002 decreased the expression of p-Akt. GSK is the abbreviation of GSK690693. AZD is the abbreviation of AZD5363. LY29 is the abbreviation of LY294002. (*P <0.05, **P <0.001, *** P <0.0001, **** P <0.00001)

To determine the functional role of Akt activation in the LRP5 knockdown-induced migration and proliferation of tongue squamous cell carcinoma, cells were treated with Akt inhibitors LY294002, GSK690693 and AZD5363. As shown in Fig. 6A-E, the treatment of CAL27 and SCC25 with GSK690693 and AZD5363 inhibited cell proliferation and migration. Among them, the effect of GSK690693 was the most obvious. As shown in Fig. 6F-I, the expression of phosphorylated Akt was reduced under the action of LY294002. Interestingly, under the action of Akt inhibitors GSK690693 and AZD5363, the expression of p-Akt increased instead. This is consistent with literature reports. It is generally believed that GSK690693 and AZD5363 block the transmission of Akt signaling, making p-Akt accumulate in cells in compensation 40-44. The phenomenon of increased levels of p-Akt upon addition of AKT inhibitors such as GSK690693 or AZD5363 may be due to a mechanism known as "feedback activation". In this scenario, AKT inhibitors suppress the activity of the AKT signaling pathway, leading cells to generate a self-regulatory response, attempting to restore the activity of the AKT signaling pathway by increasing p-Akt levels. This feedback mechanism may result in elevated p-Akt levels, despite the overall suppression of AKT signaling pathway activity. We detected p-mTOR by western blot and found that GSK690693, AZD5363, and LY294002 did inhibit the expression of p-mTOR, which is a downstream molecule of Akt signaling (Fig. 6F and 6H; Fig. S2). Therefore, it seems that the compensatory activation of Akt pathway is required for LRP5 knockdown-induced migration and proliferation of CAL27 and SCC25 cells (Graphical Abstract).

## Discussion

In this study, the researchers investigated the role of LRP5 in tongue squamous cell carcinoma (TSCC) and its potential as a therapeutic target. They found that knockdown of LRP5 in TSCC cell lines resulted in increased cell growth, which was attributed to the compensatory activation of the Akt signaling pathway. These findings suggest that inhibiting the Wnt signaling pathway alone may not be sufficient to limit the growth and progression of TSCC and that combined inhibition of both the Wnt and Akt pathways may be necessary.

In addition to its role in promoting cell survival and growth, Akt is also known to be involved in mediating resistance to various anti-tumor therapies 45. Indeed, many studies have reported that treatment with chemotherapy, radiation, or targeted therapies can lead to compensatory activation of Akt in cancer cells, which in turn promotes their survival and resistance to treatment. For instance, one study showed that treatment with the chemotherapy drug cisplatin led to upregulation of Akt in ovarian cancer cells, which was associated with increased resistance to the drug 46. Similarly, in melanoma cells, treatment with the BRAF inhibitor vemurafenib was found to induce compensatory activation of Akt, which was shown to be involved in resistance to the drug 47. Other studies have reported similar findings in various types of cancer, including breast, prostate, and pancreatic cancer. For example, in breast cancer cells, treatment with the anti-estrogen drug tamoxifen was found to induce Akt activation, which was associated with increased survival and resistance to the drug 48. In prostate cancer cells, treatment with the androgen receptor antagonist enzalutamide was found to upregulate Akt signaling, which was shown to be involved in the development of resistance to the drug 49. Together, these findings suggest that Akt plays a critical role in mediating resistance to various anti-tumor therapies, and that targeting this pathway may represent a promising strategy to overcome treatment resistance and improve patient outcomes 50. Further studies are needed to fully elucidate the molecular mechanisms underlying compensatory Akt activation in cancer cells and to identify new therapeutic targets for the treatment of cancer.

In Ren's paper 24, knocking down LRP5 or LRP6 promoted lung metastasis of breast cancer cells in a nude mouse model. They found that under conditions of reduced LRP5/LRP6, Wnt5a strongly activates the non-canonical pathway, leading to elevated p-c-jun and promoting tumor cell metastasis. Wnt3a can activate canonical Wnt signal transduction 51,52. Therefore, different tumors may rely on the canonical or non-canonical Wnt signal pathway, which may lead to different roles of LRP5 in different tumors. For example, some literature reports that Wnt5a inhibits tumor growth 53,54. Other studies have found that Wnt3a not only promotes tumor metastasis through the Wnt/β-catenin signal but also induces Rho kinase and PLC-dependent cell migration by activating PLC 52,55. It can be seen that under different ligands and receptor combinations, the canonical or non-canonical Wnt signal pathway can act synergistically or alone on tumor cells.

The findings of this study are also consistent with previous research that has shown the complexity of signaling pathways in cancer and the potential for compensatory activation of other pathways when one is inhibited. This highlights the importance of understanding the mechanisms of action of potential therapeutic targets and the need for combination therapies that target multiple pathways. The researchers used siRNA knockdown of LRP5 to investigate its role in TSCC. While this is a common method of gene knockdown, it has limitations and can result in off-target effects. The researchers acknowledged this limitation and performed transcriptome sequencing to investigate the downstream effects of LRP5 knockdown. This approach helped to validate the findings of the study and provide insights into the mechanisms of action.Overall, this study provides important insights into the role of LRP5 in TSCC and highlights the potential for combination therapies that target multiple pathways. However, further research is needed to validate these findings and investigate the potential for clinical translation.

## Supplementary Material

Supplementary figures and table.

## References

[1] Llewellyn CD, Johnson NW, Warnakulasuriya KA (2001). Risk factors for squamous cell carcinoma of the oral cavity in young people-a comprehensive literature review. Oral Oncol.

[2] Xu X, Zhang M, Xu F, Jiang S (2020). Wnt signaling in breast cancer: biological mechanisms, challenges and opportunities. Molecular cancer.

[3] Bordonaro M (2023). Oncogenic and Receptor-Mediated Wnt Signaling Influence the Sensitivity of Colonic Cells to Butyrate. J Cancer.

[4] Anastas JN, Moon RT (2013). WNT signalling pathways as therapeutic targets in cancer. Nature Reviews Cancer.

[5] Wang C, Xu X, Jin H, Liu G (2017). Nicotine may promote tongue squamous cell carcinoma progression by activating the Wnt/β-catenin and Wnt/PCP signaling pathways. Oncology Letters.

[6] Xie S-L, Fan S, Zhang S-Y, Chen W-X, Li Q-X, Pan G-K (2018). SOX8 regulates cancer stem-like properties and cisplatin-induced EMT in tongue squamous cell carcinoma by acting on the Wnt/β-catenin pathway. Int J Cancer.

[7] Goel S, Chin EN, Fakhraldeen SA, Berry SM, Beebe DJ, Alexander CM (2012). Both LRP5 and LRP6 receptors are required to respond to physiological Wnt ligands in mammary epithelial cells and fibroblasts. J Biol Chem.

[8] Badders NM, Goel S, Clark RJ, Klos KS, Kim S, Bafico A (2009). The Wnt receptor, Lrp5, is expressed by mouse mammary stem cells and is required to maintain the basal lineage. PLoS One.

[9] Lindvall C, Evans NC, Zylstra CR, Li Y, Alexander CM, Williams BO (2006). The Wnt Signaling Receptor Lrp5 Is Required for Mammary Ductal Stem Cell Activity and Wnt1-induced Tumorigenesis *. Journal of Biological Chemistry.

[10] Tsutsumi N, Hwang S, Waghray D, Hansen S, Jude KM, Wang N (2023). Structure of the Wnt-Frizzled-LRP6 initiation complex reveals the basis for coreceptor discrimination. Proceedings of the National Academy of Sciences.

[11] Ren Q, Chen J, Liu Y (2021). LRP5 and LRP6 in Wnt Signaling: Similarity and Divergence. Front Cell Dev Biol.

[12] Chen S, Bubeck D, MacDonald BT, Liang W-X, Mao J-H, Malinauskas T (2011). Structural and Functional Studies of LRP6 Ectodomain Reveal a Platform for Wnt Signaling. Dev Cell.

[13] Bhanot P, Brink M, Samos CH, Hsieh JC, Wang Y, Macke JP (1996). A new member of the frizzled family from Drosophila functions as a Wingless receptor. Nature.

[14] MacDonald BT, Tamai K, He X (2009). Wnt/β-catenin signaling: components, mechanisms, and diseases. Dev Cell.

[15] Angers S, Moon RT (2009). Proximal events in Wnt signal transduction. Nat Rev Mol Cell Biol.

[16] Li Q, Tie Y, Alu A, Ma X, Shi H (2023). Targeted therapy for head and neck cancer: signaling pathways and clinical studies. Signal Transduct Target Ther.

[17] Nie X, Liu H, Ye W, Wei X, Fan L, Ma H (2022). LRP5 promotes cancer stem cell traits and chemoresistance in colorectal cancer. J Cell Mol Med.

[18] Maubant S, Tahtouh T, Brisson A, Maire V, Némati F, Tesson B (2018). LRP5 regulates the expression of STK40, a new potential target in triple-negative breast cancers. Oncotarget.

[19] Nie X, Wang H, Wei X, Li L, Xue T, Fan L (2022). LRP5 Promotes Gastric Cancer via Activating Canonical Wnt/β-Catenin and Glycolysis Pathways. The American Journal of Pathology.

[20] Guo Y, Zi X, Koontz Z, Kim A, Xie J, Gorlick R (2007). Blocking Wnt/LRP5 signaling by a soluble receptor modulates the epithelial to mesenchymal transition and suppresses met and metalloproteinases in osteosarcoma Saos-2 cells. Journal of Orthopaedic Research.

[21] Guo Y, Rubin EM, Xie J, Zi X, Hoang BH (2008). Dominant Negative LRP5 Decreases Tumorigenicity and Metastasis of Osteosarcoma in an Animal Model. Clin Orthop Relat Res.

[22] Hong J, Xie Z, Yang Z, Yang F, Liao H, Rao S (2021). Inactivation of Wnt-LRP5 signaling suppresses the proliferation and migration of ovarian cancer cells. Transl Cancer Res.

[23] Nie X, Liu H, Ye W, Wei X, Fan L, Ma H (2022). LRP5 promotes cancer stem cell traits and chemoresistance in colorectal cancer. Journal of Cellular and Molecular Medicine.

[24] Ren D, Chen J, Li Z, Yan H, Yin Y, Wo D (2015). LRP5/6 directly bind to Frizzled and prevent Frizzled-regulated tumour metastasis. Nat Commun.

[25] Borrell-Pagès M, Romero JC, Badimon L (2014). LRP5 negatively regulates differentiation of monocytes through abrogation of Wnt signalling. J Cell Mol Med.

[26] Gonias SL, Karimi-Mostowfi N, Murray SS, Mantuano E, Gilder AS (2017). Expression of LDL receptor-related proteins (LRPs) in common solid malignancies correlates with patient survival. PLOS ONE.

[27] Zhang L, Song Y, Ling Z, Li Y, Ren X, Yang J (2019). R-spondin 2-LGR4 system regulates growth, migration and invasion, epithelial-mesenchymal transition and stem-like properties of tongue squamous cell carcinoma via Wnt/β-catenin signaling. EBioMedicine.

[28] Xiong L, Tang Y, Tang J, Liu Z, Wang X (2020). Downregulation of lncRNA HOTTIP suppresses the proliferation, migration, and invasion of oral tongue squamous cell carcinoma by regulation of HMGA2-mediated Wnt/β-catenin pathway. Cancer Biotherapy & Radiopharmaceuticals.

[29] Kina S, Kawabata-Iwakawa R, Miyamoto S, Arasaki A, Sunakawa H, Kinjo T (2021). A molecular signature of well-differentiated oral squamous cell carcinoma reveals a resistance mechanism to metronomic chemotherapy and novel therapeutic candidates. Journal of Drug Targeting.

[30] Zhao R, Wang S, Tan L, Li H, Liu J, Zhang S (2023). IGFL2-AS1 facilitates tongue squamous cell carcinoma progression via Wnt/β-catenin signaling pathway. Oral Diseases.

[31] Takebe N, Miele L, Harris PJ, Jeong W, Bando H, Kahn M (2015). Targeting Notch, Hedgehog, and Wnt pathways in cancer stem cells: clinical update. Nat Rev Clin Oncol.

[32] Smith DC, Rosen LS, Chugh R, Goldman JW, Xu L, Kapoun A (2013). First-in-human evaluation of the human monoclonal antibody vantictumab (OMP-18R5; anti-Frizzled) targeting the WNT pathway in a phase I study for patients with advanced solid tumors. J Clin Oncol.

[33] Anders S, Huber W (2010). Differential expression analysis for sequence count data. Genome Biol.

[34] Yu G, Wang L-G, Han Y, He Q-Y (2012). clusterProfiler: an R package for comparing biological themes among gene clusters. OMICS.

[35] Subramanian A, Tamayo P, Mootha VK, Mukherjee S, Ebert BL, Gillette MA (2005). Gene set enrichment analysis: A knowledge-based approach for interpreting genome-wide expression profiles. Proc Natl Acad Sci U S A.

[36] Tang Z, Li C, Kang B, Gao G, Li C, Zhang Z (2017). GEPIA: a web server for cancer and normal gene expression profiling and interactive analyses. Nucleic Acids Res.

[37] Zhang G, Li T, Tan G, Song Y, Liu Q, Wang K (2022). Identity of MMP1 and its effects on tumor progression in head and neck squamous cell carcinoma. Cancer medicine.

[38] Liu X, Yu J, Jiang LU, Wang A, Shi F, Ye H (2009). MicroRNA-222 regulates cell invasion by targeting matrix metalloproteinase 1 (MMP1) and manganese superoxide dismutase 2 (SOD2) in tongue squamous cell carcinoma cell lines. Cancer genomics & proteomics.

[39] Shimizu Y, Kondo S, Shirai A, Furukawa M, Yoshizaki T (2008). A single nucleotide polymorphism in the matrix metalloproteinase-1 and interleukin-8 gene promoter predicts poor prognosis in tongue cancer. Auris Nasus Larynx.

[40] Kostaras E, Kaserer T, Lazaro G, Heuss SF, Hussain A, Casado P (2020). A systematic molecular and pharmacologic evaluation of AKT inhibitors reveals new insight into their biological activity. Br J Cancer.

[41] Zhang Y, Zheng Y, Faheem A, Sun T, Li C, Li Z (2016). A novel AKT inhibitor, AZD5363, inhibits phosphorylation of AKT downstream molecules, and activates phosphorylation of mTOR and SMG-1 dependent on the liver cancer cell type. Oncology Letters.

[42] Choi A-R, Kim J-H, Woo YH, Cheon JH, Kim HS, Yoon S (2016). Co-treatment of LY294002 or MK-2206 with AZD5363 Attenuates AZD5363-induced Increase in the Level of Phosphorylated AKT. Anticancer Research.

[43] Li J, Davies BR, Han S, Zhou M, Bai Y, Zhang J (2013). The AKT inhibitor AZD5363 is selectively active in PI3KCA mutant gastric cancer, and sensitizes a patient-derived gastric cancer xenograft model with PTEN loss to Taxotere. Journal of Translational Medicine.

[44] Levy DS, Kahana JA, Kumar R (2009). AKT inhibitor, GSK690693, induces growth inhibition and apoptosis in acute lymphoblastic leukemia cell lines. Blood.

[45] Shan B-Q, Wang X-M, Zheng L, Han Y, Gao J, Lv M-D (2022). DCAF13 promotes breast cancer cell proliferation by ubiquitin inhibiting PERP expression. Cancer Sci.

[46] Wei X, Shi J, Lin Q, Ma X, Pang Y, Mao H (2021). Targeting ACLY Attenuates Tumor Growth and Acquired Cisplatin Resistance in Ovarian Cancer by Inhibiting the PI3K-AKT Pathway and Activating the AMPK-ROS Pathway. Front Oncol.

[47] Jiang S, Jiang T, Huang H, Chen X, Li L, Wang Z (2022). CHMFL-BMX-078, a BMX inhibitor, overcomes the resistance of melanoma to vemurafenib via inhibiting AKT pathway. Chemico-Biological Interactions.

[48] Hamadneh L, Abuarqoub R, Alhusban A, Bahader M (2020). Upregulation of PI3K/AKT/PTEN pathway is correlated with glucose and glutamine metabolic dysfunction during tamoxifen resistance development in MCF-7 cells. Sci Rep.

[49] Adelaiye-Ogala R, Gryder BE, Nguyen YTM, Alilin AN, Grayson AR, Bajwa W (2020). Targeting the PI3K/AKT Pathway Overcomes Enzalutamide Resistance by Inhibiting Induction of the Glucocorticoid Receptor. Mol Cancer Ther.

[50] Kong D, Yamori T (2008). Phosphatidylinositol 3-kinase inhibitors: promising drug candidates for cancer therapy. Cancer Sci.

[51] He S, Lu Y, Liu X, Huang X, Keller ET, Qian C-N (2015). Wnt3a: functions and implications in cancer. Chinese Journal of Cancer.

[52] Yun M-S, Kim S-E, Jeon SH, Lee J-S, Choi K-Y (2005). Both ERK and Wnt/β-catenin pathways are involved in Wnt3a-induced proliferation. Journal of Cell Science.

[53] Mikels AJ, Nusse R (2006). Purified Wnt5a protein activates or inhibits beta-catenin-TCF signaling depending on receptor context. PLoS Biol.

[54] McDonald SL, Silver A (2009). The opposing roles of Wnt-5a in cancer. Br J Cancer.

[55] Sun Z, Tang Y, Zhang Y, Fang Y, Jia J, Zeng W (2021). Joint single-cell multiomic analysis in Wnt3a induced asymmetric stem cell division. Nat Commun.

