# Supplementary material for: Knockdown of LRP5 Promotes Proliferation and Invasion of Tongue Squamous Cell Carcinoma through Compensatory Activation of Akt Signaling
Source: J Cancer. 2024 Apr 15;15(10):3215–26. doi: 10.7150/jca.93585 (PMC11064261; doi:10.7150/jca.93585)
Supplement: Supplementary file 1 — Supplementary figures and table. [file jcav15p3215s1.pdf]

Fig. S1

KEGG pathway analysis showed that the Akt signaling pathway was significantly activated after LRP5 knockdown.

Fig. S2

Western blot results showed that Akt inhibitors GSK690693 and AZD5363 significantly inhibited the expression of p-mTOR in CAL27 (A) and SCC25 (B).

Table S1: 119 up-regulated DEGs

Fig S1

A

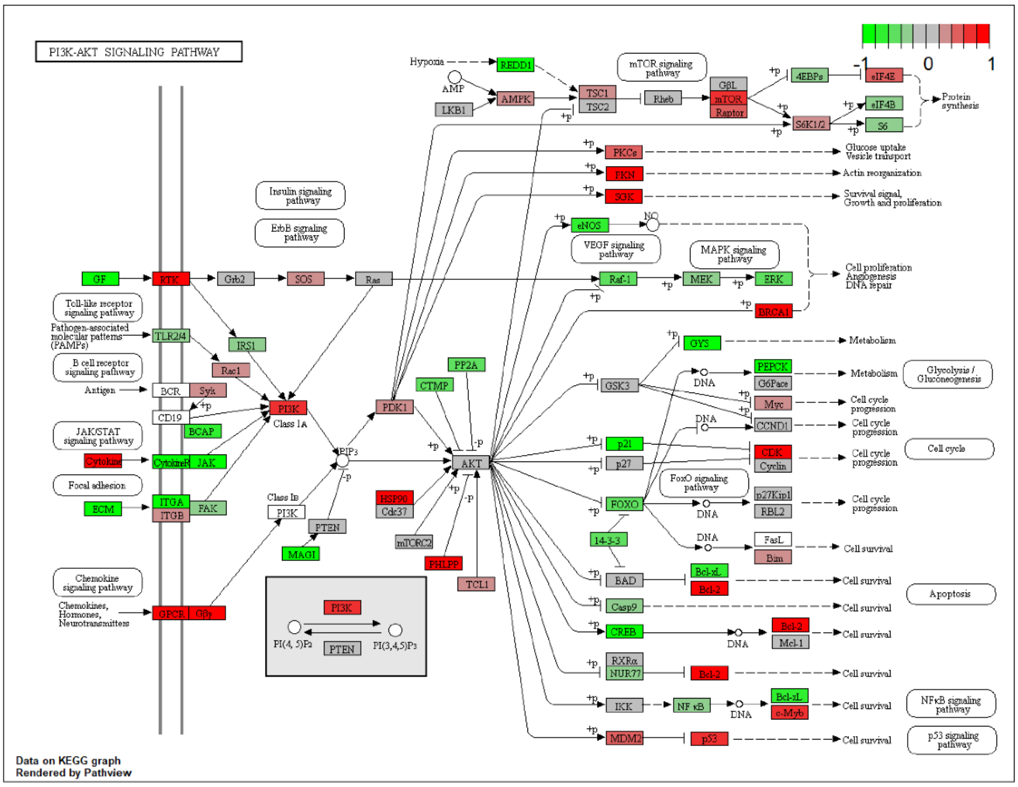

PI3K-AKT SIGNALING PATHWAY
-1 0 1
GF
RTK
Grb2
SOS
Ras
Raf-1
MEK
ERK
PI3K
PDK1
AKT
PTEN
IRS1
Rac1
TLR2/4
BCR
Syk
CD19
BCAP
JAK
ITGA
ITGB
FAK
ECM
GPCR
MAGI
PP2A
CTMP
HSP90
Cdc37
mTORC2
PHLPP
TCL1
REDD1
AMPK
LKB1
TSC1
TSC2
Rheb
GβL
mTOR
Raptor
4EBPs
eIF4E
eIF4B
S6K1/2
S6
PKCs
PKN
SGK
eNOS
BRCA1
GYS
GSK3
PEPCK
G6Pase
Myc
CCND1
p21
p27
CDK
Cyclin
FOXO
p27Kip1
RBL2
FasL
Bim
14-3-3
BAD
Bcl-xL
Bcl-2
Casp9
CREB
Mcl-1
RXRα
NUR77
IKK
NF κB
c-Myb
MDM2
p53
PI(4,5)P2
PI(3,4,5)P3
PIP3
Class IA
Class IB
Hypoxia
AMP
NO
DNA
mTOR signaling pathway
Insulin signaling pathway
ErbB signaling pathway
VEGF signaling pathway
MAPK signaling pathway
Toll-like receptor signaling pathway
Pathogen-associated molecular patterns (PAMPs)
B cell receptor signaling pathway
Antigen
JAK/STAT signaling pathway
Cytokine
Focal adhesion
Chemokine signaling pathway
Chemokines, Hormones, Neurotransmitters
FoxO signaling pathway
Glycolysis / Gluconeogenesis
Cell cycle
Apoptosis
NFκB signaling pathway
p53 signaling pathway
Protein synthesis
Glucose uptake Vesicle transport
Actin reorganization
Survival signal, Growth and proliferation
Cell proliferation Angiogenesis DNA repair
Metabolism
Cell cycle progression
Cell survival
Data on KEGG graph
Rendered by Pathview

Fig S2

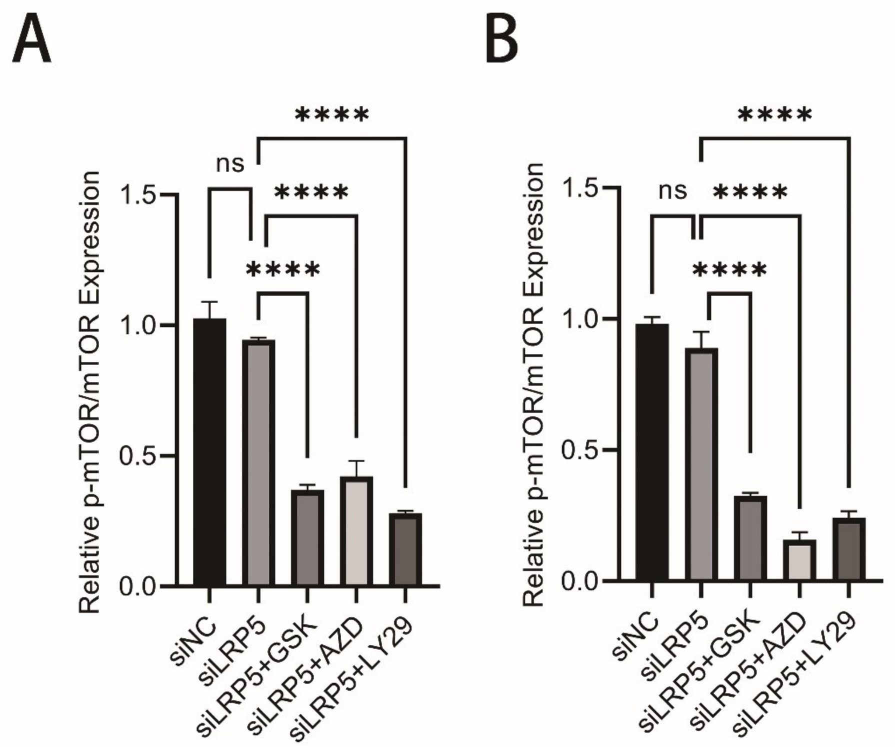
A
B
Relative p-mTOR/mTOR Expression
1.5
1.0
0.5
0.0
ns
****
siNC
siLRP5
siLRP5+GSK
siLRP5+AZD
siLRP5+LY29

Table S1: Differentially expressed genes commonly upregulated in two RNA-seqs

1	MEFV	41	LOC101927441	81	ANKRD2
2	ATP1A3	42	RAB11FIP3	82	GPER1
3	MMP13	43	ARRB1	83	DCP2
4	LY6E-DT	44	HDAC4-AS1	84	SYT8
5	NR4A2	45	LOC105370363	85	ABCB4
6	SUSD2	46	GBA	86	SLC43A2
7	CMPK2	47	EPHB3	87	SOCS1
8	LINC00260	48	LINC01132	88	METTL7A
9	LINC02043	49	GNL1	89	SEPTIN3
10	NEDD4	50	CXXC5	90	SKIDA1
11	NKD1	51	CAVIN2	91	SH3GL1P1
12	LOC105375131	52	DECR2	92	GPRASP1
13	TNNT3	53	MAPK8IP2	93	LOC107985297
14	TGFBR3L	54	SHC3	94	GJC2
15	PPM1K	55	OASL	95	CCDC194
16	IGSF1	56	IL12A	96	HERC5
17	PLCL2	57	GBP4	97	EDARADD
18	DUSP2	58	MMP1	98	RSAD2
19	BARX2	59	MMP12	99	S1PR5
20	RYR1	60	CCRL2	100	EDIL3-DT
21	EHMT2	61	KCTD20	101	TJP1P1
22	LOC107986079	62	ANKRD65	102	SHISA2
23	ARL2BP	63	ARHGDIB	103	KRT5
24	CD99	64	WTAPP1	104	RGPD8
25	TENT5C	65	MDGA1	105	NEURL1B
26	TP73	66	LOC112267871	106	TAF15
27	CXCL11	67	GRAMD1B	107	PFKFB4
28	FOXO6	68	EDIL3	108	LOC100131868
29	OTX1	69	ITGA2B	109	VDAC1P8
30	ADSS1	70	EPN3	110	IRF9
31	RTN4RL1	71	TRIM7	111	H2BC15
32	IFIT2	72	COL4A4	112	VAV3
33	STAM-AS1	73	GRIK5	113	ATP6V1B1-AS1
34	PGM2L1	74	IFI44L	114	SOX2
35	KCNQ1OT1	75	PALM3	115	CACNA1I
36	OAS1	76	IFIT1	116	NRXN2
37	ELAVL2	77	FOXL2	117	TINCR
38	MAN1C1	78	OAS2	118	IFIT3
39	HCAR2	79	LOC105374128	119	IQGAP2
40	HMGA2-AS1	80	NAP1L4P1		
